# Opioid Overdose Prevention Programs Providing Naloxone to Laypersons — United States, 2014

**Published:** 2015-06-19

**Authors:** Eliza Wheeler, T. Stephen Jones, Michael K. Gilbert, Peter J. Davidson

**Affiliations:** 1 Drug Overdose Prevention and Education (DOPE) Project, Harm Reduction Coalition, Oakland, California; 2 T. Stephen Jones Public Health Consulting, Florence, Massachusetts; 3 T. H. Chan School of Public Health, Harvard University, Boston, Massachusetts; 4 University of California, San Diego, California

Drug overdose deaths in the United States have more than doubled since 1999 (1). During 2013, 43,982 drug overdose deaths (unintentional, intentional [suicide or homicide], or undetermined intent) were reported (1). Among these, 16,235 (37%) were associated with prescription opioid analgesics (e.g., oxycodone and hydrocodone) and 8,257 (19%) with heroin (2). For many years, community-based programs have offered opioid overdose prevention services to laypersons who might witness an overdose, including persons who use drugs, their families and friends, and service providers. Since 1996, an increasing number of programs provide laypersons with training and kits containing the opioid antagonist naloxone hydrochloride (naloxone) to reverse the potentially fatal respiratory depression caused by heroin and other opioids (3). In July 2014, the Harm Reduction Coalition (HRC), a national advocacy and capacity-building organization, surveyed 140 managers of organizations in the United States known to provide naloxone kits to laypersons. Managers at 136 organizations completed the survey, reporting on the amount of naloxone distributed, overdose reversals by bystanders, and other program data for 644 sites that were providing nalox-one kits to laypersons as of June 2014. From 1996 through June 2014, surveyed organizations provided naloxone kits to 152,283 laypersons and received reports of 26,463 overdose reversals. Providing opioid overdose training and naloxone kits to laypersons who might witness an opioid overdose can help reduce opioid overdose mortality.

Since 2008, HRC has maintained a database of organizations providing naloxone kits to laypersons. The Opioid Safety and Naloxone Network is a national network of naloxone experts, program administrators, and advocates. Before the survey, HRC staff polled network participants for information on any new organizations providing naloxone kits to laypersons that should be included in the survey. In July 2014, HRC e-mailed a link to an online survey to managers of 140 organizations known to provide naloxone kits to laypersons. These organizations included public health departments, pharmacies, health care facilities, substance use treatment facilities, and community-based organizations providing services to persons who use drugs, including current or former opioid (heroin or pharmaceutical) users, and other potential witnesses to overdoses. Law enforcement organizations, emergency medical services, and other professional first responders using naloxone were not included in this survey.

The survey included questions about the year the organization began operating; the numbers of sites or local programs providing naloxone kits; the number of persons trained in overdose prevention and provided naloxone kits; and the number of reports of overdose reversals (administration of naloxone by a trained layperson in the event of an overdose) (4), as well as whether the reports were based on program data or were estimates. The survey also asked about the naloxone formulations currently provided in kits, models for training and providing naloxone kits, funding sources, and any difficulties obtaining naloxone. To obtain data for a recent full calendar year, organizations providing naloxone kits during calendar year 2013 were asked to provide specific data for that year, including numbers of persons provided naloxone kits, reversals reported, and naloxone vials provided; characteristics of persons who received naloxone kits (e.g., persons who use drugs, friends and family members, service providers); characteristics of persons reporting overdose reversals; and the drugs involved in reported overdose reversals. HRC staff used follow-up e-mails and telephone calls to encourage participation and clarify responses.

Managers from 136 (97.1%) organizations completed the survey, including those from 84 community-based organizations, 18 health care facilities, 10 Veterans Administration health care systems, 18 state or local health departments, and six pharmacies. Half of the responding organizations began operating during January 2013–June 2014 (Figure 1). Respondents provided reports for 644 local opioid overdose prevention sites that provide naloxone kits, located in 30 states and the District of Columbia (DC) (Figure 2). Thirty-eight respondents provided consolidated data for multiple local sites providing naloxone kits. Some organizations estimated responses; for example, one health department estimated the number of laypersons receiving naloxone kits on the basis of the number of kits distributed to local sites. Three state health departments (Massachusetts, New Mexico, and New York) oversee operations of statewide naloxone programs, with 334 local sites (51.9% of the 644 local sites).

From 1996, when the first organization began providing naloxone, through June 2014, the 136 responding organizations reported providing training and naloxone kits to 152,283 laypersons (range = 1–36,450; median = 100; mean = 1,120).* The 109 organizations that collect reports of reversals documented 26,463 overdose reversals (range = 0–5,430; median = 9; mean = 243).†

During 2013, 93 organizations reported distributing or prescribing naloxone to 37,920 laypersons (range = 0–9,000; median = 75; mean = 407.7).§ The 68 (50%) organizations that collect reports of reversals documented 8,032 overdose reversals (range = 0–2,079; median = 10; mean = 118.1).¶

Ninety-three organizations collected information on the characteristics of laypersons who were provided naloxone kits. Laypersons who received naloxone kits were characterized as persons who use drugs (81.6%); friends and family members (11.7%); service providers (3.3%); or unknown (3.4%).** Sixty-eight organizations provided information about lay-persons who reported administering naloxone, characterizing them as persons who use drugs (82.8%); friends and family members (9.6%); service providers (0.2%); or unknown (7.4%).†† Forty-two organizations collected information from laypersons about the drugs that appeared to be involved in the reversed overdoses; heroin was involved in 81.6% and prescription opioids in 14.1%.§§

Various program models were used by organizations to provide naloxone to laypersons, including distribution of naloxone kits by trained nonmedical staff or volunteers under a standing order (60 [44.1%]), by medical staff (49 [36.0%]), prescriptions written by a medical provider and filled at a pharmacy (39 [28.7%]), pharmacists dispensing directly via collaborative practice agreements and other mechanisms (12 [8.8%]), and other protocols (19 [14.0%]). Thirty-three organizations used more than one model.

During 2013, 90 (66.2%) of the 136 organizations reported distributing 140,053 naloxone vials, including refills (range = 1–53,200; median = 179.5; mean = 1,556.1).¶¶ Three respondents whose organizations were operational in 2013 did not report on the number of vials because they furnished prescriptions to be filled at a pharmacy. The remaining 43 organizations indicated that they were not yet providing naloxone kits during 2013. Sixty-nine respondents (50.7%) reported their organization provided only injectable naloxone, 51 (37.5%) provided only intranasal naloxone, and 16 (11.8%) provided both injectable and intranasal naloxone.*** A total of 111,602 vials (79.7%) of injectable naloxone (21.4% 10 mL and 58.1% 1 mL) and 28,446 (20.3%) vials of intranasal naloxone were provided to laypersons. Organizations were characterized as small, medium, large, or very large, on the basis of the number of naloxone vials distributed during 2013. The 11 large and very large organizations provided naloxone to 28,604 laypersons, representing 75.4% of all 2013 recipients (Table). Forty (29.4%) organizations reported difficulties maintaining an adequate supply of naloxone, and 73 (53.7%) reported inadequate resources to sustain or expand their organization’s efforts to disseminate naloxone kits.

## Discussion

Organizations that provide naloxone kits to laypersons have expanded substantially since a similar survey in 2010 (5), reflecting a 183% (from 48 to 136) increase in the number of responding organizations; a 243% (from 188 to 644) increase in the number of local sites providing naloxone; a 187% (from 53,032 to 152,283) increase in the number of laypersons provided naloxone kits; a 160% (from 10,171 to 26,463) increase in the number of reversals reported; and a 94% (from 16 to 30) increase in states (including DC) with at least one organization providing naloxone. Half of the responding organizations began operating during January 2013–June 2014. Although early adopters of naloxone kit provision were mainly syringe exchanges, other programs, including substance use treatment facilities, Veterans Administration health care systems, primary care clinics, and pharmacies have started providing naloxone to laypersons.

Providing naloxone kits to laypersons reduces overdose deaths (4), is safe (3), and is cost-effective (6). U.S. and international health organizations recommend providing naloxone kits to laypersons who might witness an opioid overdose (3,7); to patients in substance use treatment programs (3,7,8); to persons leaving prison and jail (3,7,8); and as a component of responsible opioid prescribing (8).

Although the number of organizations providing naloxone kits to laypersons is increasing, in 2013, 20 states had no such organization, and nine had less than one layperson per 100,000 population who had received a naloxone kit. Among these 29 states with minimal or no access to naloxone kits for laypersons, 11 had age-adjusted 2013 drug overdose death rates higher than the national median (2).

Some organizations reported information on the laypersons receiving naloxone kits (N = 99 organizations), using naloxone in overdose reversals (N = 68), and the drugs that appeared to have caused the overdose (N = 42). Persons who use drugs accounted for 81.6% of laypersons who received naloxone kits; they also performed the majority (82.8%) of reported overdose reversals. A majority (81.6%) of the overdoses that were reversed involved heroin, indicating that organizations are reaching laypersons who witness heroin overdoses. A study of a community-based naloxone program in San Francisco also found that persons who use drugs play a major role in reversing heroin overdoses (9). Additional interventions are needed to reach persons who may witness prescription opioid analgesic overdoses, which account for nearly twice as many deaths as heroin overdoses.

Forty (29.4%) respondents reported that their organization has experienced problems obtaining naloxone. Prices of intranasal naloxone more than doubled in the second half of 2014 (10) and Opioid Safety and Naloxone Network members report that cost increases are reducing the quantity of naloxone purchased and provided to laypersons (Matt Curtis, VOCAL NY, personal communication, 2015).

The findings in this report are subject to at least four limitations. First, despite extensive knowledge of naloxone distribution programs by the Harm Reduction Coalition and Opioid Safety and Naloxone Network, organizations providing naloxone kits are increasing rapidly and some might not yet be known to HRC and therefore, might not be included in the survey, which may underestimate the impact of these programs. Second, survey responses are based on unconfirmed reports from organizations providing naloxone kits. Third, some reports provided by organizations are based on estimates. These three limitations could result in either under or over-reporting of persons provided naloxone kits. Finally, the numbers of overdose reversals likely were under-reported, because some sites, such as pharmacies, do not collect reversal reports.

Organizations providing naloxone kits to laypersons receive many reports of overdose reversals and can reach large numbers of potential overdose bystanders. Comprehensive prevention measures that include teaching laypersons how to respond to overdoses and administer naloxone might help prevent opioid drug overdose deaths. This report suggests that many programs reach persons who witness heroin-related overdoses; additional methods are needed to provide naloxone kits to persons who might witness prescription opioid analgesic overdoses.

**Summary**

What is already known on this topic?

Drug overdose deaths in the United States have more than doubled since 1999, reaching a total of 43,982 in 2013. Heroin and prescription opioids are major causes of drug overdose deaths. Naloxone is the standard medication used for reversal of the potentially fatal respiratory depression caused by opioid overdose.

What is added by this report?

From 1996 through June 2014, a total of 644 local sites in 30 states and the District of Columbia reported providing naloxone kits to 152,283 laypersons and receiving reports of 26,463 drug overdose reversals using naloxone from 1996 through June 2014. Most laypersons who reported using the kits to reverse an overdose were persons who use drugs, and many of the reported reversals involved heroin overdoses. Medical clinics and pharmacies have started providing naloxone kits to laypersons, and the reported number of organizations providing kits almost doubled from January 2013 through June 2014.

What are the implications for public health practice?

Organizations training and providing naloxone kits to laypersons can reach large numbers of potential overdose witnesses and result in many reported overdose reversals. Comprehensive prevention measures that include teaching laypersons how to respond to overdoses and administer naloxone prevent opioid-related drug overdose deaths. Additional methods are needed to provide naloxone kits to persons who might witness prescription opioid analgesic overdoses.

## Figures and Tables

**FIGURE 1:**
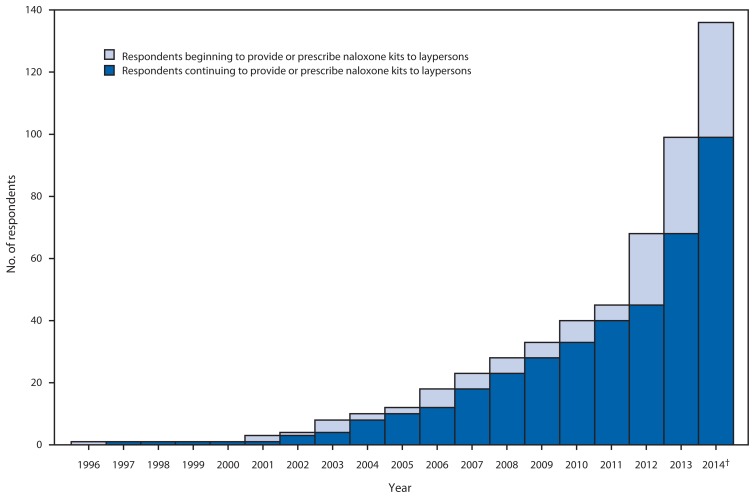
Number of survey respondents reporting beginning or continuing to provide naloxone kits to laypersons, by year — United States, 1996–June 2014*^†^ * Results of a survey conducted in July 2014 by the Harm Reduction Coalition, in which 136 organizations reported 644 local sites where laypersons were trained to recognize an opioid drug overdose and provided or prescribed naloxone kits. ^†^ As of June 2014.

**FIGURE 2:**
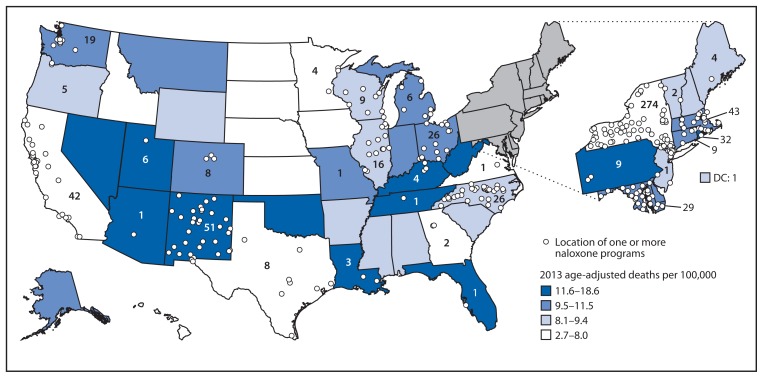
Number* and location of local drug overdose prevention programs providing naloxone to laypersons, as of June 2014, and age-adjusted rates^†^ of drug overdose deaths^§^ in 2013 — United States * Total N = 644; numbers on map indicate the total number of programs within each state. ^†^ Per 100,000 population. ^§^ CDC, National Center for Health Statistics; Compressed Mortality File 1999–2013 on CDC WONDER Online Database, released January 2015.

**TABLE:** Reported number of laypersons receiving or prescribed naloxone kits, overdose reversals, and opioid overdose prevention programs, by survey respondent program size — United States, 1996–June 2014

					Calendar year 2013				1996—June 2014			
				
	Respondents		Sites		Laypersons received/prescribed kits†		Opioid overdose reversals§		Laypersons received/prescribed kits¶		Opioid overdose reversals**	
						
Category (by size)*	No.	(%)	No.	(%)	No.	(%)	No.	(%)	No.	(%)	No.	(%)
Small (<100)	84	(61.8)	154	(23.9)	1,709	(4.5)	134	(1.7)	7,867	(5.2)	641	(2.4)
Medium (101–1,000)	41	(30.1)	129	(20.0)	7,607	(20.1)	1,351	(16.8)	19,239	(12.6)	4,414	(16.7)
Large (1,001–10,000)	7	(5.1)	62	(9.6)	6,117	(16.1)	4,329	(53.9)	29,099	(19.1)	11,807	(44.6)
Very large (>10,000)	4	(2.9)	299	(46.4)	22,487	(59.3)	2,218	(27.6)	96,078	(63.1)	9,601	(36.3)
**Total**	**136**	**(100.0)**	**644**	**(100.0)**	**37,920**	**(100.0)**	**8,032**	**(100.0)**	**152,283**	**(100.0)**	**26,463**	**(100.0)**

* Based on reported number of vials of naloxone provided during 2013.

† Calendar year 2013 information provided by 93 survey respondents distributing kits/prescribing naloxone during that year, with 36 estimating (6,483 [17.1%] persons) and 57 based on program data (31,437 [82.9%]).

§ Sixty-eight of 93 respondents distributing kits/prescribing naloxone in 2013 provided information on reported reversals, with 13 estimating (659 [8.2%] reversals) and 55 based on program data (7,373 [91.8%]).

¶ Estimated by 57 survey respondents (55,201 [36.2%] persons) and 79 based on program data (97,082 [63.8%]).

** Program began in 1996; as of June 2014, 109 respondents distributing kits/prescribing naloxone provided information on reported reversals, with 28 estimating (5,245 [19.8%] reversals) and 81 based on program data (21,218 [80.2%]).

## Footnotes

* Estimated by 57 respondents (55,201 [36.2%] laypersons) and based on program data for 79 (97,082 [36.8%]).

† Estimated by 28 respondents (5,245 [19.8%] reversals) and based on program data for 81 (21,218 [80.2%]).

§ Estimated by 36 respondents (6,483 [17.1%] laypersons) and based on program data for 57 (31,437 [82.9%]).

¶ Estimated by 13 respondents (659 [8.2%] reversals) and based on program data for 55 (7,373 [91.8%]).

** Estimated by 48 (51.6%) respondents and based on program data for 45 (48.4%).

†† Estimated by 26 (38.2%) respondents and based on program data for 42 (61.8%).

§§ Estimated by 18 (42.9%) respondents and based on program data for 24 (57.1%).

¶¶ Estimated by 37 survey respondents (31,838 [22.7%] vials) and based on program data for 53 (108,215 [77.2%]).

*** Organizations provide laypersons with naloxone for injection and/or for intranasal administration. Injectable naloxone is distributed in multi-dose (10 mL) and single-dose (1 mL) vials with concentrations of 0.4 mg/mL. Intranasal naloxone is distributed in single-dose 2 mL vials with concentration of 1 mg/mL that are adapted for intranasal use with a mucosal atomizer.
